# Robust land surface temperature record for north China over the past 21,000 years

**DOI:** 10.1126/sciadv.adj4800

**Published:** 2024-02-21

**Authors:** Jingjing Guo, Martin Ziegler, Niko Wanders, Mike Vreeken, Qiuzhen Yin, Hao Lu, Louise Fuchs, Jibao Dong, Youbin Sun, Francien Peterse

**Affiliations:** ^1^ Department of Earth Sciences, Utrecht University, 3584 CB Utrecht, Netherlands.; ^2^ Department of Physical Geography, Utrecht University, 3584 CB Utrecht, Netherlands.; ^3^ Earth and Climate Research Center, Earth and Life Institute, Université catholique de Louvain, Louvain-la-Neuve, Belgium.; ^4^ State Key Laboratory of Loess and Quaternary Geology, Institute of Earth Environment, Chinese Academy of Sciences, Xi’an 710061, China.

## Abstract

Numerous proxy reconstructions have provided general insight into late Quaternary East Asian Monsoon variability. However, challenges persist in precisely assessing absolute temperature impacts on proxy variations. Here, we use two independent paleothermometers, based on bacterial membrane lipids and clumped isotopes of snail shells, in the same section of the western Chinese Loess Plateau to establish a robust land surface temperature record spanning the past approximately 21,000 years. Our independent temperature records consistently reveal (i) similar land surface temperatures between the Last Glacial Maximum and late Holocene and (ii) a gradual cooling Holocene, which contrasts with the climate model predictions. We propose that changes in soil moisture availability over the deglaciation modulates the land surface temperature recorded by the proxies. A land surface energy partitioning model confirms this mechanism, suggesting that effects of soil moisture availability should be properly considered when comparing proxy records with climate model outputs.

## Teaser

A robust 21,000-year temperature record for north China suggests a strong impact of moisture availability on surface temperatures.

## INTRODUCTION

The East Asian Monsoon (EAM) is among the strongest monsoon systems on Earth. The EAM is driven by temperature contrasts between the Pacific Ocean and the Asian mainland, which is further enhanced by the thermal and dynamic effects of the Tibet Plateau (*1*). The EAM consists of a warm and wet summer monsoon (EASM) and a cold and dry winter monsoon (EAWM). The EASM is led by the northward migration of the intertropical convergence zone and transports moisture from the west Pacific and Indian Oceans to East Asia, thereby driving precipitation on the continent. The EAWM results from pressure gradients between the Siberian High and the Aleutian Low over the north Pacific Ocean. The northwesterly wind transports aeolian dust from the Gobi and sandy deserts in northwest China and Southern Mongolia, leading to the buildup of the Chinese Loess Plateau (CLP) (*2*). As one of the best paleoclimate archives on land, Chinese loess captures glacial-interglacial EAM variability in the alternating sequences of loess and paleosols. The loess-paleosol sequences also record millennial-scale climate events, which are transmitted from North Atlantic through the Westerlies (*3*). Variations in past EAM climate are routinely reconstructed based on proxies of grain size (GS) and magnetic susceptibility (MagSus) from loess-paleosol sequences of the CLP. The MagSus is generally interpreted as a proxy of EASM intensity relying on the mechanism that soil pedogenesis is more pronounced under warm and wet conditions, with higher MagSus reflecting a stronger EASM (*4*). The GS has been used as a proxy of winter monsoon strength linked through dust transport, with coarser GS corresponding to a stronger EAWM (*5*, *6*).

Insights into Quaternary EAM history are furthermore obtained from, e.g., the oxygen isotope composition (δ^18^O) of cave speleothems from southeast China (*7*, *8*), pollen assemblages (*9*, *10*) or Si/Al ratios of lake sediment records (*11*), and the δ^18^O, Mg/Ca ratio and assemblages of foraminifera in marine sediments (*12*, *13*). However, most of these proxies are influenced by both temperature and precipitation, resulting in the long-lasting confusion and debate in the understanding of orbital-scale EAM climate variability and dynamics. A classic issue is the so-called “Chinese 100-ka problem” (*14*). Specifically, loess MagSus records are dominated by the ~100-ka (thousand years) cycle, implying a major control of Northern Hemisphere (NH) ice volume on the EASM (*15*), while speleothem records show a dominant ~20-ka cycle, implying a major control of precession on the EASM (*7*, *8*). However, rather than EASM intensity, speleothem δ^18^O records represent a combined signal of moisture source, transport pathway, and precipitation intensity of the EASM (*14*, *16*). Similarly, the MagSus of loess-paleosol sequences can also be affected by temperature and sedimentation rate (*17*, *18*). As such, the ambiguity in the climatic interpretation of these proxies restricts the validation of climate model outputs (*19*, *20*). The different patterns reflected by these EASM indicators suggest that internal climate feedbacks may also play a role (*14*, *21*). Therefore, an accurate reconstruction of temperature and precipitation is essential to further constrain the dynamics and forcing mechanisms of the EAM. In particular, the response of EASM precipitation to global warming requires a robust evaluation of the temperature effect on these EAM proxy records. So far, most studies have focused on the reconstruction of monsoon precipitation (*8*, *22*). However, the evolution of land temperature, regulating the strength of the EAM through the land-sea temperature contrast, and its relationship with monsoon precipitation are less explored.

The absence of such temperature records was for a long time attributed to a lack of quantitative temperature proxies for the terrestrial realm. Over the past two decades, two continental paleothermometers have emerged from two different fields. The first paleothermometer is based on branched glycerol dialkyl glycerol tetraethers (brGDGTs; fig. S1), membrane lipids that are synthesized by certain soil bacteria (*23*, *24*). The number of methyl branches (4 to 6) that is attached to the alkyl backbone of 5-methyl brGDGTs, quantified in the MBT′_5Me_ (see Materials and Methods) (*25*), shows an empirical relationship with the mean annual air temperature (MAAT) in set of global surface soils. Although the analytical error on brGDGT measurements is very small (<0.02°C), the temperature calibration shows a relatively large uncertainty (~4°C, see Materials and Methods), which derives from the large heterogeneity in soils in the global dataset, especially at mid-latitudes (*26*). Further uncertainty originates from the expression of the temperature relationship of brGDGT producers, which can occur through physiological adaptations in the cell membrane, as well as, or complementary to shifts in the composition of their producers (*27*). Nevertheless, the physiological basis for the relationship between temperature and MBT′_5Me_ has recently been confirmed by molecular dynamics simulations (*28*) and experiments with the one acidobacterial strain that has so far been found to produce brGDGTs in culture (*29*–*31*). The brGDGTs have been successfully used to generate continuous temperature records for several sections on the CLP (*32*–*39*). Notably, some brGDGT records seem to suggest that deglacial warming occurred before the increase in solar insolation, which remains hard to explain, but could also be related to age model uncertainties (*32*–*35*). In addition, the brGDGT-based temperatures are generally higher than the expected MAAT, which has been interpreted as a bias toward growing season (*32*, *36*, *40*), or that brGDGTs reflect land surface temperature rather than air temperature (*35*, *41*), based on the assumption that the microbes that produce brGDGTs live in the soil and better thrive under warm and wet conditions. The Bayesian model that calibrates brGDGT distributions to mean monthly temperatures above freezing (BayMBT_0_) has a lower uncertainty than earlier calibrations that use MAAT (~2°C lower than the Bayesian model, BayMBT, and ~1°C lower than the linear regression) (*25*, *26*). In addition, a recent global temperature data compilation revealed consistent differences between the 2-m air temperature and surface soil temperature, which can be up to 10°C under specific hydroclimate conditions (*42*), potentially explaining part of the remaining scatter in the calibration.

The second paleothermometer is based on the occurrence of “clumping” of two heavy rare isotopes (^13^C and ^18^O) in carbonate minerals, expressed as ∆_47_, where thermodynamics dictate that more clumping occurs at lower temperatures (*43*, *44*). This method has recently been applied to a stalagmite from northern China to reconstruct temperature changes during the penultimate deglaciation (*45*). On the CLP, carbonate can be found in land snail shells that are widely distributed and well preserved. Snail assemblages as such can be used to infer changes in EAM climate based on the distinct optimal living conditions of different snail species on the modern CLP (*46*). In addition, the δ^18^O values of land snail shells (δ^18^O_shell_) are used to reconstruct past EASM climate, although the δ^18^O_shell_ actually relates to both temperature and precipitation (*47*, *48*). The ∆_47_ yields the absolute temperature at which the carbonate was formed, independent of the δ^18^O composition of the source water (*49*). However, available studies so far indicate that snail shells can generate ∆_47_-based temperatures that are higher than MAAT, as well as the mean air temperature during the snail’s active season (*50*–*52*). This has been attributed to ecophysiological adaptations of snails, as are expressed in their shell morphology, coloration, and the preference for warm growth conditions (*52*, *53*). In contrast to the brGDGT paleothermometer, the Δ_47_-based temperature calibration has a relatively small error (<0.1°C) (*54*, *55*) but requires large sample size and multiple replicate analyses of the same sample to overcome intrinsic analytical uncertainties (*56*). As a result, currently available ∆_47_-based temperature reconstructions for the CLP are mostly limited to a single data point per time interval, thereby disregarding the timing and rate of past temperature change that is reflected in brGDGT-based temperature records (*51*, *52*). Hence, to generate robust paleotemperatures, it is important to combine these two independent tools.

**Fig. 1.**
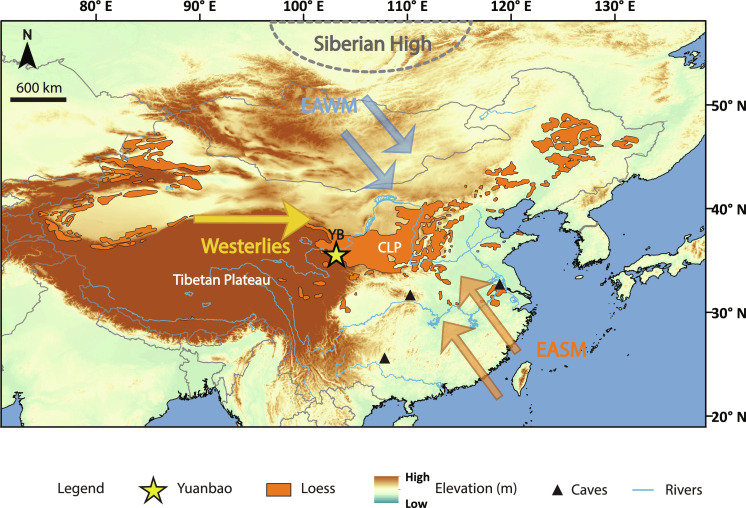
Map of the study site and main wind patterns. The study site, Yuanbao, is indicated with a yellow star. The locations of the Hulu, Sanbao, and Dongge caves from which the speleothem δ^18^O record is derived are indicated by black triangles. CLP, Chinese Loess Plateau; EASM, East Asian Summer Monsoon; EAWM, East Asian Winter Monsoon.

Here, we apply both methods in parallel on the upper 6 m of the Yuanbao loess section to reconstruct absolute temperature records for north China since the last deglaciation. Yuanbao section is located on the western CLP at the modern reach of the EASM, EAWM, and mid-latitude Westerlies (*57*), making the site sensitive to (past) changes in the relative monsoon strength and associated climate conditions of these wind systems (Fig. 1). Furthermore, the Yuanbao section is characterized by a high sedimentation rate (~18 and ~71 cm ka^−1^ for paleosol and loess layers, respectively; fig. S2), enabling the generation of a high-resolution temperature record. The analysis of both brGDGTs and clumped isotopes in fossil snail shells of *Pupilla muscorum* and *Vallonia tenera* species (fig. S3) allows us to validate our continuous, high-resolution brGDGT-based temperature record with a relatively large uncertainty, with well-constrained Δ_47_-based temperatures in a lower resolution but with a small uncertainty. We also aim to resolve the supposed seasonality in the temperature signal reflected by brGDGTs by using snail ecological constraints and place the timing of deglacial temperature evolution in the context of EASM strengthening reflected by the MagSus record from the same section. Last, we use a land surface energy partitioning model to assess the effect of soil moisture availability on our temperature proxies. Together, this leads to a robust record of land surface temperature during the summer monsoon season in north China over the last deglaciation.

## RESULTS

### Temperature reconstruction

**Fig. 2.**
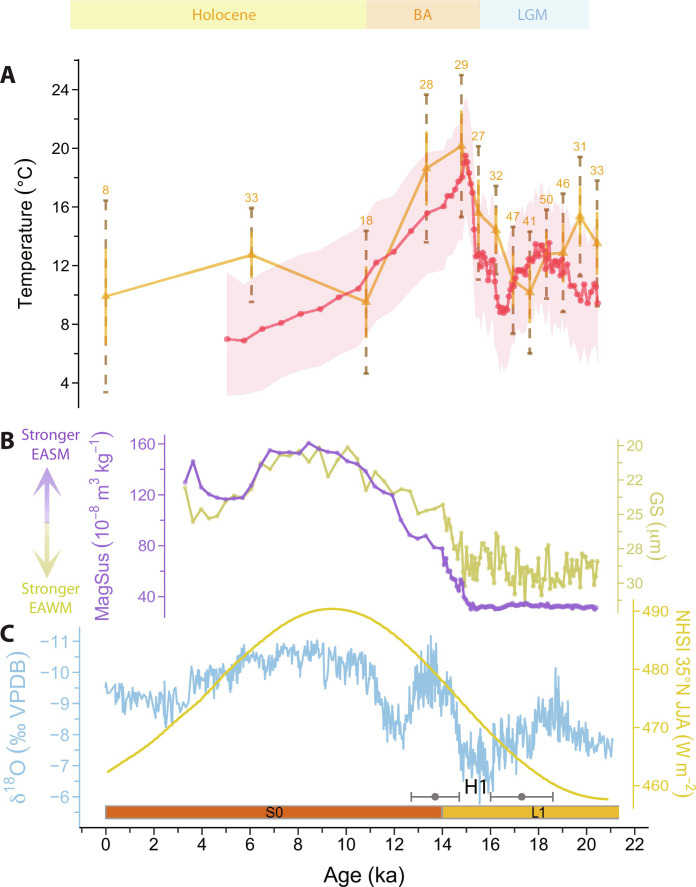
Reconstructed land surface temperature change in north China over the last deglaciation. (**A**) Temperature based on brGDGTs (red line, shaded areas represent 1σ uncertainties) and clumped isotopes of snail shells (yellow line, 1σ and 2σ uncertainties indicated by solid yellow and dashed brown lines, respectively; numbers indicate replicate measurements for each sample). (**B**) Grain size (GS; dark yellow) representing strength of EAWM and magnetic susceptibility (MagSus; purple) indicating EASM intensity at Yuanbao. (**C**) δ^18^O of stacked speleothem records from Hulu/Sanbao/Dongge caves (*8*) and Northern Hemisphere Summer Insolation (NHSI) at 35°N (June, July, and August) (*58*). OSL dates and their uncertainty are shown by gray bars above the *x* axis (17.3 ± 1.3 ka and 13.7 ± 1 ka). Brown and yellow bars above the *x* axis represent paleosol and loess layers, respectively. H1, Heinrich event 1; BA, Bølling-Allerød; LGM, Last Glacial Maximum.

BrGDGTs were analyzed in 89 loess samples (each integrating 5 cm) at 10-cm intervals for the Holocene paleosol S0 and 5- or 10-cm intervals for the last glacial loess L1, resulting in an average resolution of the brGDGT record of ~520 years for S0 and ~80 years for L1 (see Materials and Methods for the chronology). Their relative distributions translate into temperatures between ~7° and ~20°C (Fig. 2A). These temperatures are in line with those reported for an earlier brGDGT-based record from Yuanbao (*37*), although that record has a much lower resolution (*n* = 16 for the same time interval) and is based on an analytical method that does not separate brGDGT isomers with a methylation on position C-5 or C-6 of the alkyl backbone. Because the 6-methyl brGDGTs strongly relate to soil pH rather than temperature (*25*), this implies that the earlier record actually represents a mixed signal of temperature and (precipitation-induced changes in) soil pH.

Our temperature record, based only on the temperature-sensitive 5-methyl brGDGTs, varies between ~9° and ~14°C from 21 to 17 ka, where the minimum of ~9°C coincides with Heinrich event 1 (H1). Subsequently, brGDGT-based temperatures rapidly and substantially increase (~10°C) during the Bølling-Allerød (BA) to reach a peak of ~20°C around 15 ka. After that, temperature abruptly drops down and then gradually decreases until ~7°C during the late Holocene. The brGDGTs reflect similar temperatures within proxy error for the late Holocene (i.e., ~6 ka) and the Last Glacial Maximum (LGM).

The temperatures derived from the clumped isotope composition of snail shells obtained from the same loess-paleosol material that was used for brGDGT analysis vary between 10° and 20°C (Fig. 2A). The resolution of the clumped isotope record (*n* = 13) allows us to validate not only the absolute temperatures reflected by the brGDGTs but also the trends and timing of changes in this record. Notably, the ∆_47_-based temperature record mirrors that of the brGDGTs and also reflects similar temperatures for the late Holocene and the LGM, as well as the large temperature increase during the BA (~10°C), all within the error margins of both proxies (Fig. 2A). Together, our brGDGT and clumped isotope-based records show that temperature change not only generally follows Northern Hemisphere Summer Insolation (NHSI) over the deglaciation (*58*) but also contains millennial scale events like H1 and the BA, showing a remarkable similarity with the changes in the Chinese speleothem δ^18^O record within the age uncertainties (Fig. 2C) (*8*). In addition, although a Holocene Thermal Maximum is not expressed at Yuanbao, the gradual cooling during the Holocene is consistent with the global trends in terrestrial and marine proxy records rather than the warming simulated by climate models (*59*, *60*), although the degree of cooling at our site is larger than that in global compilations (Fig. 2A).

### GS and MagSus records

The GS and MagSus records from the same loess section remain stable until ~15 ka and then display a gradual decrease and increase until ~9 ka (Fig. 2B). Notably, the trends and timing of the GS and MagSus records are substantially different from those of the temperature records. This is particularly clear at ~17 ka when temperature starts to increase, whereas the GS and MagSus records only start to change at ~15 ka (Fig. 2, A and B). A second deviation occurs during the Holocene, where both temperature records show a gradual cooling from ~15 ka onward, while the GS and MagSus records continue to respectively decrease and increase from the early to the mid-Holocene (Fig. 2, A and B). This distinct behavior of temperature and GS and MagSus records is also visible on the larger CLP, as practically all currently available brGDGT-based temperature records have reached their maximum and start to decrease well before the GS and MagSus records reach their peak values during the Holocene (*32*–*35*, *38*).

## DISCUSSION

### Influence of growing season length on proxy-based temperatures

The reconstructed temperature for the late Holocene and the LGM is similar in both brGDGT and clumped isotope-based records. However, global records (*61*) and model outputs (*62*) indicate that Holocene mean annual surface temperatures were ~5° to 6°C higher than during the LGM. For the brGDGT record, our temperature estimates are generated using the BayMBT_0_ calibration that is based on the average temperature of months above freezing (MAF) (*26*). The similar temperatures for the late Holocene and the LGM may in part be explained by the fact that microbes require a certain amount of moisture and a temperature threshold to live and grow, whereas winters and glacial periods on the CLP are arid and cold (*2*). This means that the production of brGDGTs and their temperature signal would be biased toward the wetter and warmer conditions that occur during summers (*32*, *36*, *40*). A pronounced bias toward summer temperatures in brGDGT-based temperatures is also observed for modern surface soils with a temperature seasonality of >20°C (*26*). The present climate conditions on the western CLP, where large seasonal fluctuations in both temperature (~25°C) and precipitation (~80% of the 500 mm year^−1^ falls during summer) occur, would facilitate the introduction of such a warm season bias.

Similarly, snails only build their shells during their active period, i.e., when the environmental conditions are suitable. Snails are sensitive to temperature and moisture changes (*63*) and enter hibernation when temperature and relative humidity structurally drop below a certain threshold (*64*, *65*). For one of the species analyzed in this study (*V. tenera*), these thresholds are not reported in the literature, but for the species of *Pupilla*, it is known that they are not active at temperatures <10°C (*66*). Clumped isotope measurements on the modern shells of *P. muscorum* at Yuanbao generate a temperature of 10° ± 3°C (1σ; 10° ± 7°C, 2σ; *n* = 8, fewer replicates resulting from limited material introduce a relatively high uncertainty, see Materials and Methods for details). This would correspond to a growing season from April to October (average mean air temperature for these months is ~12°C; fig. S4). These months all receive on average >32 mm month^−1^ precipitation and relative humidity is >57% (fig. S4), suggesting that this species needs at least this level of moisture availability, next to mean temperatures >10°C, to be active. In addition, culturing experiments (*67*, *68*) and earlier modern land snail studies (*50*, *53*) have shown that ∆_47_-based temperatures of snail shells reflect their growing season temperature rather than the MAAT, and that ecophysiological modifications can also influence ∆_47_-based temperatures (*50*, *51*, *53*). Thus, the strong coherence between ∆_47_ and brGDGT-based temperatures indicates that both records reflect the temperature of the wet and warm summer monsoon season in north China over the past 21 thousand years (kyr). The similar reconstructed temperatures for the late Holocene and the LGM in both proxy records imply that temperatures during the summer monsoon season were comparable during these two time intervals; however, changes in the length and severity of the winters that would influence MAAT are likely not recorded.

### Influence of local climate on proxy-based temperatures

Besides changes in growing season length, a change in wind patterns may also influence LGM temperatures. A climate modeling experiment has indicated that the presence of ice sheets can introduce nonlinear wave patterns in the prevailing winds (*69*). While the ice sheets lead to cooling in the mid-high latitudes, the modified wind pattern can reduce this effect at certain locations by blocking winds from the north. As such, these winds could partly explain the unexpectedly similar Holocene and LGM temperatures reconstructed by the proxies in our record (Fig. 2A).

Furthermore, the high elevation of Yuanbao [2177 m above sea level (masl)] and its proximity to the Tibetan Plateau (TP) and nearby mountain glaciers may contribute to the relatively lower temperatures during the Holocene. Both morphostratigraphy and a coupled mass balance and ice flow model suggest that glaciers on the northeast and eastern part of the TP advanced during the early Holocene due to the increase in precipitation brought by the EASM at this time (*70*, *71*). The growth of the glaciers on the TP is estimated to have induced a cooling of ~0.5° to 1.5°C on the regional climate (*70*, *72*) and may thus have also affected the climate at Yuanbao at this time.

### Influence of soil moisture availability on proxy-based temperatures

To further assess the reliability of our temperature records, we consider the possible influence of soil moisture availability on both temperature proxies. A recent study compared in situ soil temperatures with the air temperature at 2 m above ground level and found that surface soils in cold and dry regions can be substantially (up to 10°C) warmer than the above air, while surface soils in warm and wet areas are slightly cooler due to the different partitioning of incoming radiation (*42*). The offset between soil and air temperature is attributed to the difference in heat capacity between dry air and water, which results in that arid soils warm up faster than wet soils (*73*). This also implies that organisms thriving in the soil, such as the brGDGT producers, and on the soil surface, like the snails, can experience abiotic conditions that are substantially different from those at 2 m above the ground. In this scenario, both proxies are thus assumed to reflect the temperature of the soil surface rather than that of the air.

The GS and MagSus records indicate that the LGM was dominated by the EAWM, which is characterized by arid and cold conditions and thus limited soil moisture (Fig. 2B). The low heat capacity of the soil during this time would enhance the heat absorption supplied by solar radiation, and result in high soil surface temperatures relative to those of the air. The warming comes to a halt at ~15 ka, exactly the moment that GS and MagSus proxies start to change and indicate a weakening of the EAWM (finer GS) and strengthening of the EASM (higher MagSus), and thus a change in soil moisture conditions (Fig. 2, A and B). The increase in soil moisture availability and thereby its heat capacity has a dampening effect on the soil temperature from ~15 ka onward. As a result, the reconstructed temperatures are lower than what could be expected from the increase in solar energy (Fig. 2, A and C). Hence, the cooling trend from the early to mid-Holocene that is reflected by our two temperature records may in part be the result of the change in soil moisture availability rather than an actual change in air temperature, in addition to the decrease in NHSI during the mid-Holocene (Fig. 2C). This same trend has also been observed in other sections on the CLP, where the maximum temperature is reached before that of MagSus, and the decrease in temperature exactly coincidences with the increase in MagSus (*32*, *34*, *35*).

Another interesting feature in our records is that the onset warming leads the onset of changes in MagSus by ~2 kyr (Fig. 2, A and B). The temporal offset between brGDGT and MagSus records from the CLP has been observed before (*32*–*36*), but the consistency of both temperature proxies now confirms that this lag is not an artifact of the brGDGT paleothermometer and that temperature change is indeed decoupled from the MagSus signal. This offset has previously been explained not only by the presence of NH ice sheets blocking atmospheric teleconnections that force the EASM (*32*, *36*) but also as the result of a change in vegetation cover and corresponding shading effect during glacial-interglacial transitions, where sparse vegetation during glacials would allow the still dry and relatively exposed soil to warm up rapidly with increasing solar radiation (*35*, *74*, *75*). However, vegetation reconstructions based on plant leaf waxes indicate that the central CLP was always covered by vegetation even during glacials (*76*, *77*), although vegetation shifted from mainly C3 woody vegetation during glacials to C3 nonwoody plants during interglacials (*78*). Furthermore, vegetation cover is ultimately shaped by soil moisture availability (*79*) especially in arid and semi-arid areas (*80*), suggesting that this latter parameter may be key in explaining the temperature signal represented by our proxies. Regardless, a decrease in shading effects due to a reduced vegetation cover will presumably amplify offsets between air and land surface temperature. For example, a more open landscape due to limited soil moisture availability before ~15 ka could explain the unexpectedly large amplitude of the rapid BA warming (~10°C within 2 kyr) reflected in both temperature records as a result of the low heat capacity of dry soil.

**Fig. 3.**
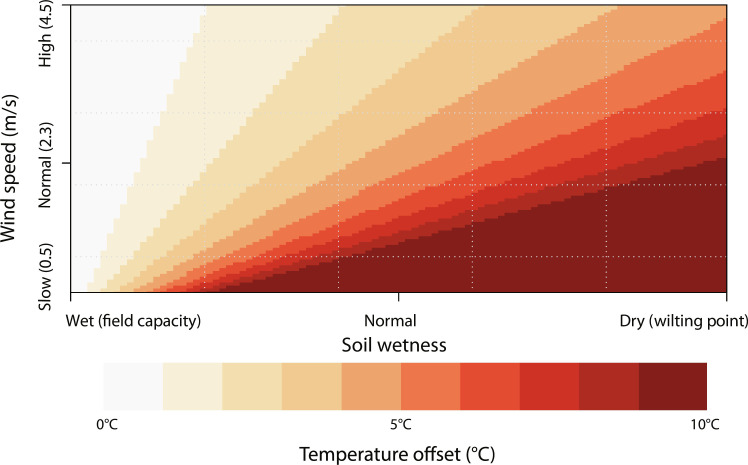
Modeled daytime temperature offsets between soil surface and air temperature. Color gradients indicate the temperature difference between the soil surface and 2-m air temperature. Changes in soil moisture availability can explain temperature offset up to 10°C.

To further test the presumed influence of soil moisture availability on land surface temperature, we modeled the offset of temperature at the surface versus that of the air at 2 m under varying soil moisture and wind conditions on the CLP while keeping a constant vegetation cover. The model outputs indicate that under arid and less windy conditions, the soil temperature can be up to 10°C higher than the air temperature (Fig. 3). Although stronger winds during glacials could dampen the difference between soil and air temperature, the model indicates that soil temperature is still ~5°C higher than that of air (Fig. 3). Similarly, soil temperatures can also be lower than the air temperature under wet conditions as experienced during interglacials. Given the impacts of soil moisture availability on the vegetation cover, the presence of dynamic vegetation cover under different soil moisture conditions will likely contribute to the more pronounced temperature variations in our record (fig. S5). Regardless, these outcomes support that the large degree of BA warming and the subsequent abrupt decrease in reconstructed temperatures at ~15 ka can be attributed to variations in soil moisture availability.

Our parallel application of two independent paleothermometers provides high confidence in the reconstructed temperature records. On the basis of the ecological constraints of the snails used for clumped isotope analysis, we infer that both proxies reflect warm season land surface temperatures and that soil moisture availability exerts an important influence on these two temperature proxies through its influence on the heat capacity of the soil, which is confirmed by land surface energy partitioning model results. The resulting record confirms a long-term cooling trend during warm seasons in East Asia during the Holocene. Hence, the temperature sensitivity to soil moisture availability should be properly considered in comparisons between proxy data and simulated air temperatures from climate models.

## MATERIALS AND METHODS

### Study site and sampling

The Yuanbao loess-paleosol sequence (35.63°N, 103.17°E) is situated near Linxia city on the western edge of the CLP (Fig. 1). The mean annual air temperature (MAAT) in Linxia is ~7°C (China Meteorological Administration, https://data.cma.cn/en; fig. S4). This translates into a MAAT of ~6°C at the elevation of the sample site (2177 masl, based on a lapse rate of 0.6°C 100 m^−1^) with temperatures of around −6°C in winter (December–February), ~11°C during the MAF, and ~12°C during the growing season (April–October). The mean annual precipitation (MAP) is 500 mm year^−1^, of which ~80% falls between May and September, and the annual evaporation is 1300 mm (*37*, *81*). Samples were collected from the Yuanbao section in August 2019 with 5-cm intervals from a 6-m-deep pit, covering the S0 (Holocene paleosol) and part of L1 (Last Glacial loess). Fossil snail shells were isolated from ~20 kg of loess collected at 10-cm intervals that were wet sieved in the field.

### MagSus and GS analysis

MagSus and GS were analyzed at 5-cm resolution (*n* = 120) at the Institute of Earth Environment, Chinese Academy of Sciences. Low-frequency mass MagSus was measured with a Bartington Instruments MS2 meter. After removal of organic matter (10 ml, 10% H_2_O_2_) and carbonate (10 ml, 10% HCl), the GS distributions were determined using a Malvern 2000 laser diffraction instrument. Measurement uncertainty was <10% for MagSus and <2% for mean GS.

### Chronology

The chronology of the Yuanbao section is described by Fuchs *et al.* (*82*). Briefly, the chronology is established through the alignment of the generated mean GS and MagSus records to those obtained from a drill core collected nearby (35.15°N, 103.63°E, 2200 masl) in 2017 (*81*). The obtained age model furthermore aligns with the optically stimulated luminescence (OSL) dates from a nearby loess-paleosol sequence CH02/02 at Yuanbao (35.64°N, 103.15°E, 2177 masl) (*83*). The chronology indicates that the upper 6 m of our Yuanbao profile covers the past approximately 21,000 years, corresponding with a sedimentation rate of 18 cm ka^−1^ for S0 and 71 cm ka^−1^ for L1 on average (fig. S2).

### BrGDGT extraction and analysis

To avoid anthropogenic perturbation, the upper 30 cm of the loess section was excluded from analysis. In total, 89 samples were analyzed for brGDGTs, covering the Holocene paleosol S0 (30 to 200 cm) in 10-cm resolution, and last glacial loess L1 in 5- to 10-cm resolution (200 to 500 cm in 5-cm resolution and 500 to 600 cm in 10-cm resolution). For each depth, ~30 g of loess was freeze-dried and homogenized, after which it was extracted with dichloromethane (DCM):methanol (MeOH) (9:1, v/v) using a Milestone ETHOS X microwave extractor at 70°C to obtain a total lipid extract (TLE). The TLEs were filtered to remove remaining sediment using preextracted filter paper (Whatman grade 42 Ashless Filter Paper, 55 mm diameter) and dried under a N_2_ stream. After the addition of a known amount of C_46_ glycerol trialkyl glycerol tetraether internal standard (*84*), the TLEs were separated into apolar and polar fractions by passing them over an activated Al_2_O_3_ column using hexane:DCM (9:1, v/v) and DCM:MeOH (1:1, v/v), respectively. The polar fraction, which contains the brGDGTs, was evaporated to dryness under a gentle N_2_ stream. After this, the samples were prepared for further analysis by redissolving them in a hexane:isopropanol (99:1, v/v) mixture and filtration through a 0.45-μm polytetrafluoroethylene (PTFE) filter. The brGDGTs were analyzed on an Agilent 1260 Infinity ultrahigh-performance liquid chromatography coupled to an Agilent 6130 single quadrupole mass spectrometer at Utrecht University (*85*). Quantitation was achieved by peak area integration of the [M + H]^+^ ions in Chemstation software B.04.03.

Temperature changes reflected by the brGDGTs were quantified using the MBT′_5Me_ index based on their relative abundances (*25*, *86*, *87*)

$${\text{MBT}\prime }_{5\mathrm{Me}}=(\mathrm{Ia}+\mathrm{Ib}+\mathrm{Ic})/(\mathrm{Ia}+\mathrm{Ib}+\mathrm{Ic}+\mathrm{IIa}+\mathrm{IIb}+\mathrm{IIc}+\mathrm{IIIa})$$ (1)

in which roman numerals refer to the molecular structures of brGDGTs in fig. S1.

MBT′_5Me_ index values were subsequently translated into mean air temperatures for MAF using the BayMBT_0_ model (*25*, *26*), and a prior mean of 10.9°C (modern mean air temperature of all months >0°C at Yuanbao) and a prior SD of 15°C were chosen as input for the BayMBT_0_ model. The root mean square error (RMSE) on the calibration is 3.8°C (*26*). This calibration model is based on the assumption that the bacteria produced brGDGTs are active when the ambient environment is above 0°C, and thus accounts for the effects of temperature seasonality on brGDGT production (*26*). Although the calibration uncertainty is relatively high in mid-latitude regions (*26*), this uncertainty is mainly systematic and introduced by soil heterogeneity that will thus be reduced when the MBT′_5Me_ index is applied on a local scale, like here on the Yuanbao section (*32*). All temperatures are reported with a 68% confidence interval (1σ) combining analytical and calibration uncertainties. The analytical uncertainty is based on monitoring regular runs of a laboratory standard with a known GDGT composition, and accounts <0.02°C.

### Clumped isotope measurement and analysis

Two species of fossil snail shells were picked under a Keyence VHX-2000 Super Resolution Digital Microscope based on the snail classification in the CLP (*88*). *P. muscorum* and *V. tenera* were present throughout the studied interval but had relatively higher abundances in the loess layer (fig. S3). Both species belong to the cold-aridiphilous mollusk group that thrives under cold and dry climate conditions (*88*). The fossil snail shells were rinsed with distilled water and further cleaned four to five times (~30 s per time) in an ultrasonic bath to remove any particles attached to the tests. The shells were oven-dried at 40°C for 24 hours and then crushed into homogeneous powder with an agate mortar and pestle in preparation for clumped isotope analysis. The clumped isotope composition of snail shells was determined using a MAT 253 Plus mass spectrometer (Thermo Fisher Scientific) coupled to a Kiel IV carbonate device for sample gas preparation. Around 80 to 100 μg of calcite ETH standards (ETH-1, ETH-2, and ETH-3) and sample aliquots were reacted with 103% phosphoric acid at 70°C and run in a ratio around 1:1 (*89*). The released CO_2_ gas was cleaned from water and organic matter by two liquid nitrogen (LN_2_) traps kept at −40°C, with a Porapak Type Q trap embedded in the second LN_2_ trap. The purified gas was measured in long integration dual inlet mode with integration time of 400 s, followed by a CO_2_ working gas (δ^13^C = −2.82 ‰, δ^18^O = −4.67 ‰), which is used for pressure baseline correction. To generate precise temperature estimates (*56*), >20 aliquots were run for each sample, except for the modern (*n* = 8) and ~10 ka (*n* = 18) samples, of which limited material was available.

Δ_47_ raw values were transferred into the InterCarb-Carbon Dioxide Equilibrium Scale (I-CDES) using an empirical transfer function based on the measurements of standards (ETH-1, ETH-2, and ETH-3) within a moving window of 100 standards before and after the target sample, and their accepted I-CDES values (*54*). Although Δ_47_-temperature transfer functions have been developed for specific land snail species (e.g., *Bradybaena* and *Cathaica*) (*90*), these calibrations are not available for the snail species used here. Because the snail shells are mainly composed of aragonite (*91*) and a recent study confirmed that clumped isotope calibrations predominantly based on calcite samples can also be applied on aragonite samples (*92*), we here used Eq. 2 that is valid for the temperature range <28°C for ∆_47_ temperature reconstruction (*55*, *93*)

$${∆}_{47}=\left(0.0397\pm 0.0011\right)\times 10^{6}/T^{2}+\left(0.1518\pm 0.0128\right)$$ (2)

Uncertainty of the regression is incorporated and propagated in the final reported confidence interval following the same procedure reported in other studies (*94*). Most of the Δ_47_-based temperature uncertainty derives from the analytical uncertainty, while the calibration uncertainty only accounts for a relatively small part. The ∆_47_-based temperatures of different snail species from the same samples are not significantly different (*P* = 0.7) and, hence, were combined for the final temperature records.

### Physical modeling of soil moisture variability

To evaluate the absolute temperature offsets between soil surface and 2-m air temperature under different soil moisture conditions, a land surface energy partitioning model was derived that allocated the incoming radiation into latent (λ*E*) and sensible heat (*H*) (Eq. 3). Only the situation with positive incoming radiation is considered (i.e., daytime scenario). We assumed that all the incoming radiation was used for latent heat under wet conditions, while all energy was used in sensible heat under dry conditions

Net incoming radiation (Q)=λE+H (3)

We have used the equation below for the estimation of land-air temperature offsets (*95*)

$$T(z)=T_{0}-H/\left(\rho \times C_{p}\times C_{\mathrm{HN}}\times u\right)$$ (4)

in which *T*_0_ indicates the aerodynamic surface temperature, *H* is the sensible heat flux from surface to air, ρ is the density of the air (1.27 kg m^−3^), *C*_*p*_ is the air specific heat at constant pressure [1005 J/(kg·K)] (*96*), *u* is the wind speed based on modern observations on the CLP (*97*), *C*_*HN*_ is the bulk transfer coefficients in the neutral state (*95*), and

$$C_{\mathrm{HN}}=k^{2}/{\left[\ln\left(z/z_{h}\right)\right]}^{2}$$ (5)

where *k* is von Karman constant (0.4), *z* is the dependent height (2 m for air temperature in our study), and *z*_*h*_ is a roughness parameter (0.1 m in our study). Only sensible heat (*H*) was changed as a result of the wet (*H* = 0) or dry (*H* = 490 W m^−2^ based on the maximum NHSI over the past 21 kyr) condition. The other variables were kept constant and close to the current conditions for vegetation and resulting roughness.

For this experiment, the vegetation height was set to 10 cm (*z_h_* = 0.1 m) based on field observations and plant leaf wax records from other high elevation sections on the CLP, indicating that the current vegetation types were also dominant in the past 21 kyr (*76*, *78*). The modern average summer wind speed on the CLP is 2.3 m s^−1^ (*97*), based on which a maximum and minimum wind speed of 0.5 and 4.5 m s^−1^ were assumed. This information was used to compute the temperature difference between the land surface and air temperature at 2 m. By varying the soil wetness, the incoming radiation is portioned in different fractions to sensible heat and evapotranspiration, resulting in changes in the land surface–air temperature offset. We assumed that on an average day the ground heat flux is negligible compared to the other fluxes. Within this assessment, potential changes in vegetation cover are also considered by adjusting the sensible heat flux (*H*) to 75 and 50% (368 and 245 W m^−2^, respectively, mimicking 25 and 50% vegetation cover) and the vegetation height (adjusting *z_h_* to 1 and 4 m, respectively). Model outputs for different vegetation cover scenarios are given in fig. S5. The land surface energy partitioning model was undertaken in R software (*98*) based on Eqs. 4 and 5, and the R script is in the Supplementary Materials.
