# Supplementary material for: Robust land surface temperature record for north China over the past 21,000 years
Source: Sci Adv. 2024 Feb 21;10(8):eadj4800. doi: 10.1126/sciadv.adj4800 (PMC10881045; doi:10.1126/sciadv.adj4800)
Supplement: Supplementary file 1 — Figs. S1 to S5 Legends for data S1 to S3 [file sciadv.adj4800_sm.pdf]

Science Advances
AAAS

Supplementary Materials for

Robust land surface temperature record for north China over the past 21,000 years

Jingjing Guo *et al.*

Corresponding author: Jingjing Guo, j.guo@uu.nl

Sci. Adv. **10**, eadj4800 (2024)
DOI: 10.1126/sciadv.adj4800

The PDF file includes:

Figs. S1 to S5
Legends for data S1 to S3

Other Supplementary Material for this manuscript includes the following:

Data S1 to S3

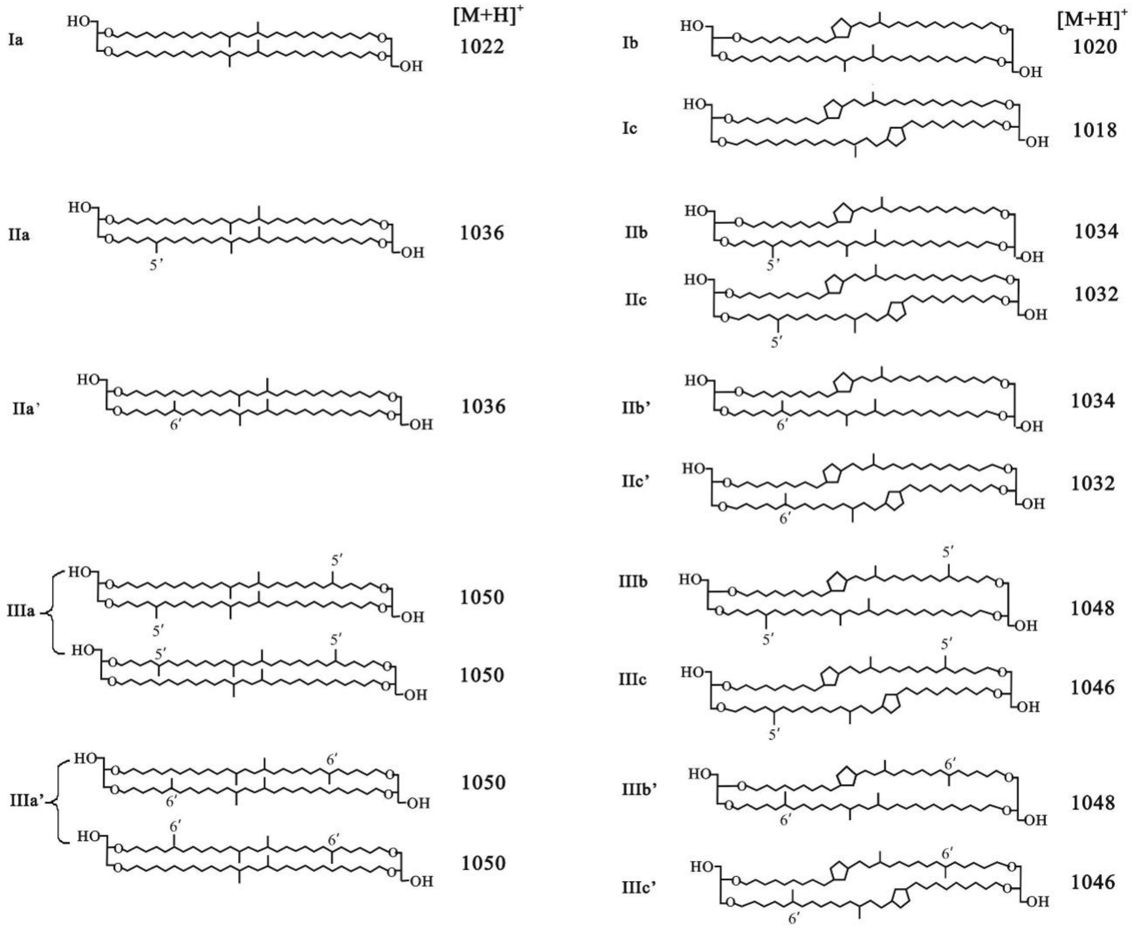

Fig. S1.

Molecular structures of 5-methyl and 6-methyl branched GDGTs. The 6-methyl brGDGTs are represented by a prime symbol. The structures of penta- and hexamethylated brGDGTs with cyclopentane moiety(ies) IIb', IIc', IIIb', IIIc' are tentative.

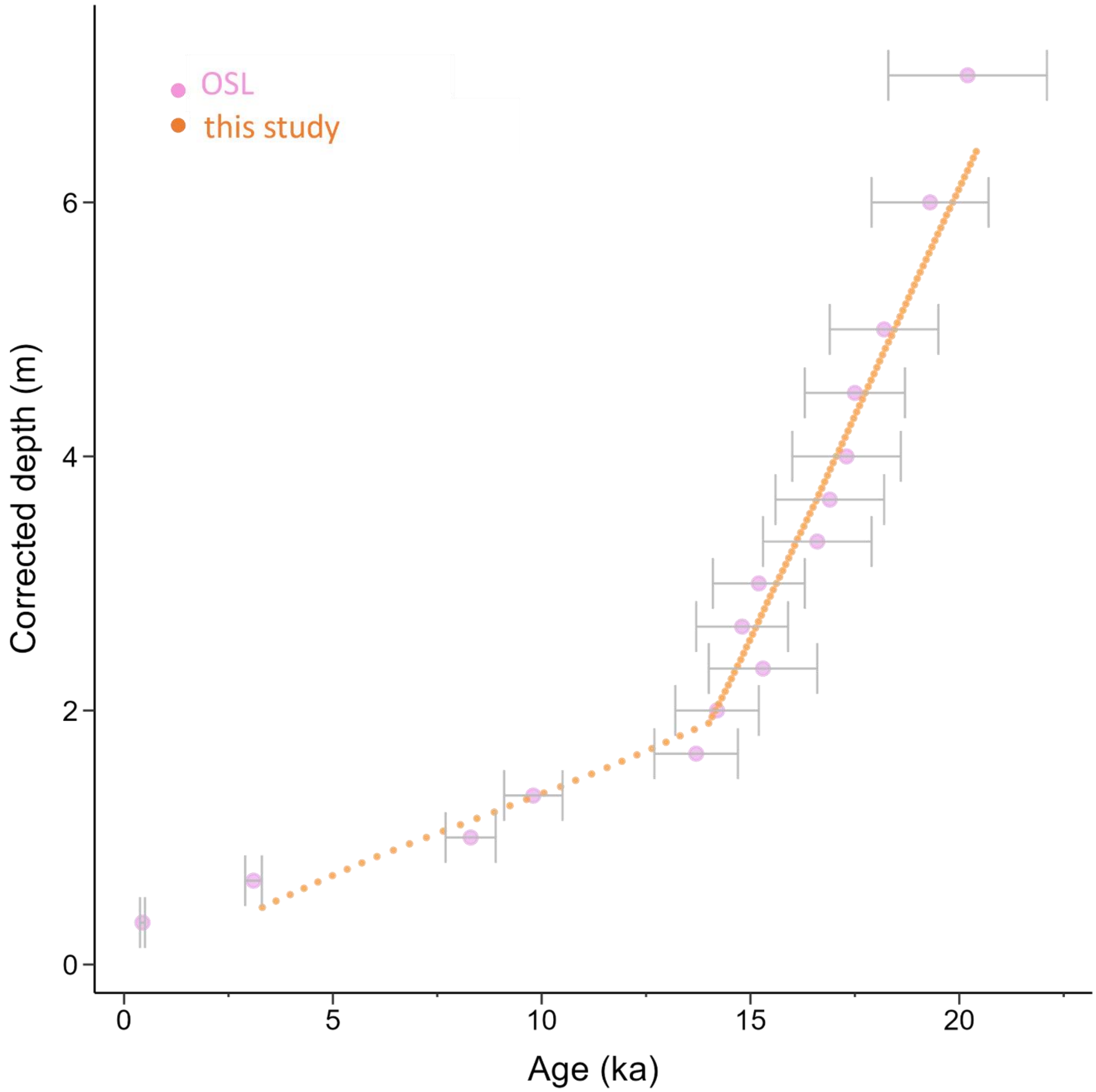

Fig. S2.

Sedimentation rate at Yuanbao. Age versus depth plot in which the orange dots are samples in this study, and the light pink dots with gray error bars are from a nearby loess-paleosol sequence at Yuanbao (CH02/02) based on Optically Stimulated Luminescence (OSL) dating (See Methods for details).

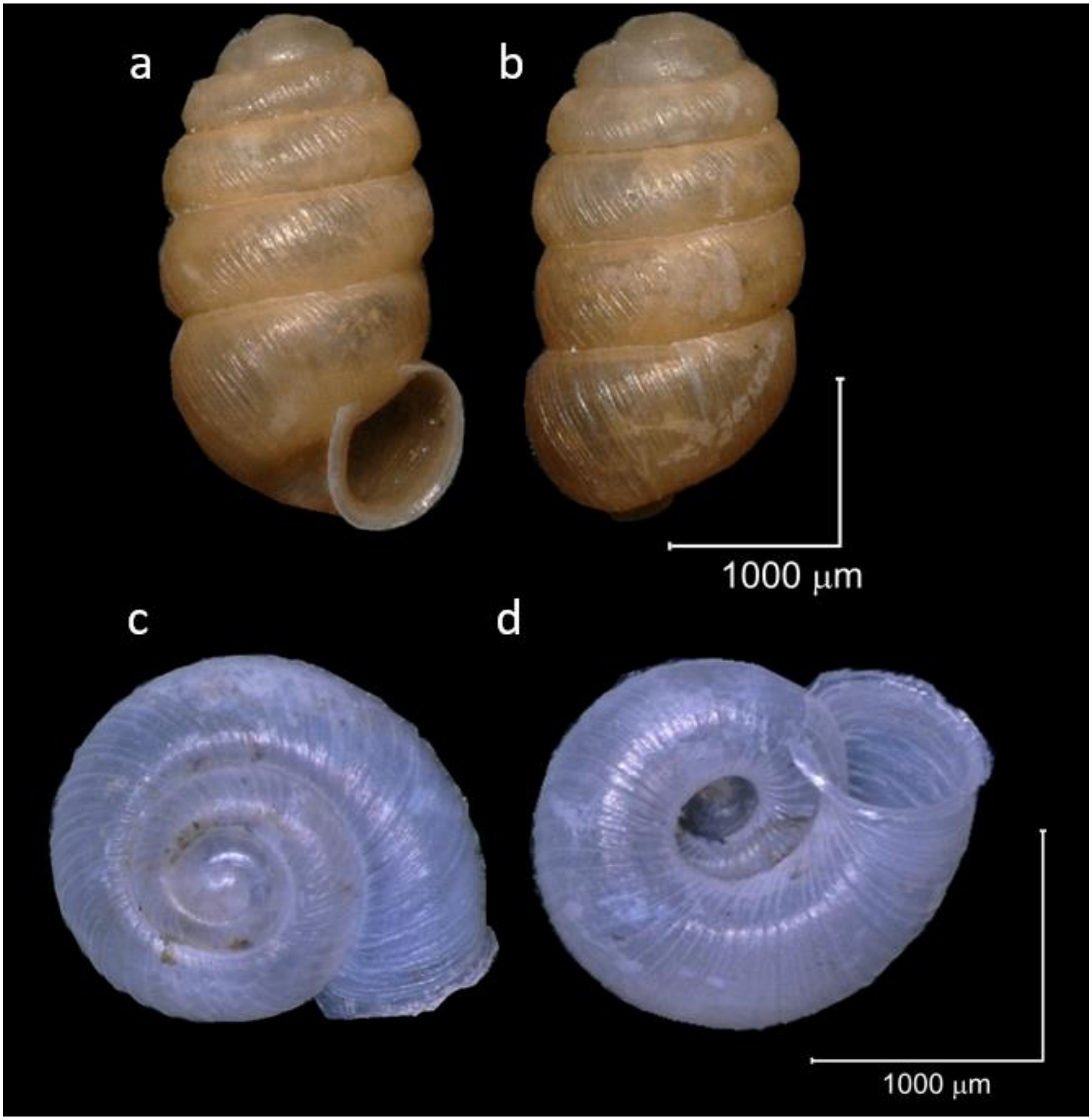

Fig. S3.

Snail shells used for clumped isotope measurements. Two snail shells that were retrieved from 500 cm depth/~19 ka from the Yuanbao section on the Chinese Loess Plateau. (a, b) Lateral and dorsal views of *Pupilla muscorum*; (c, d) Lateral and umbilical views of *Vollonia tenera*.

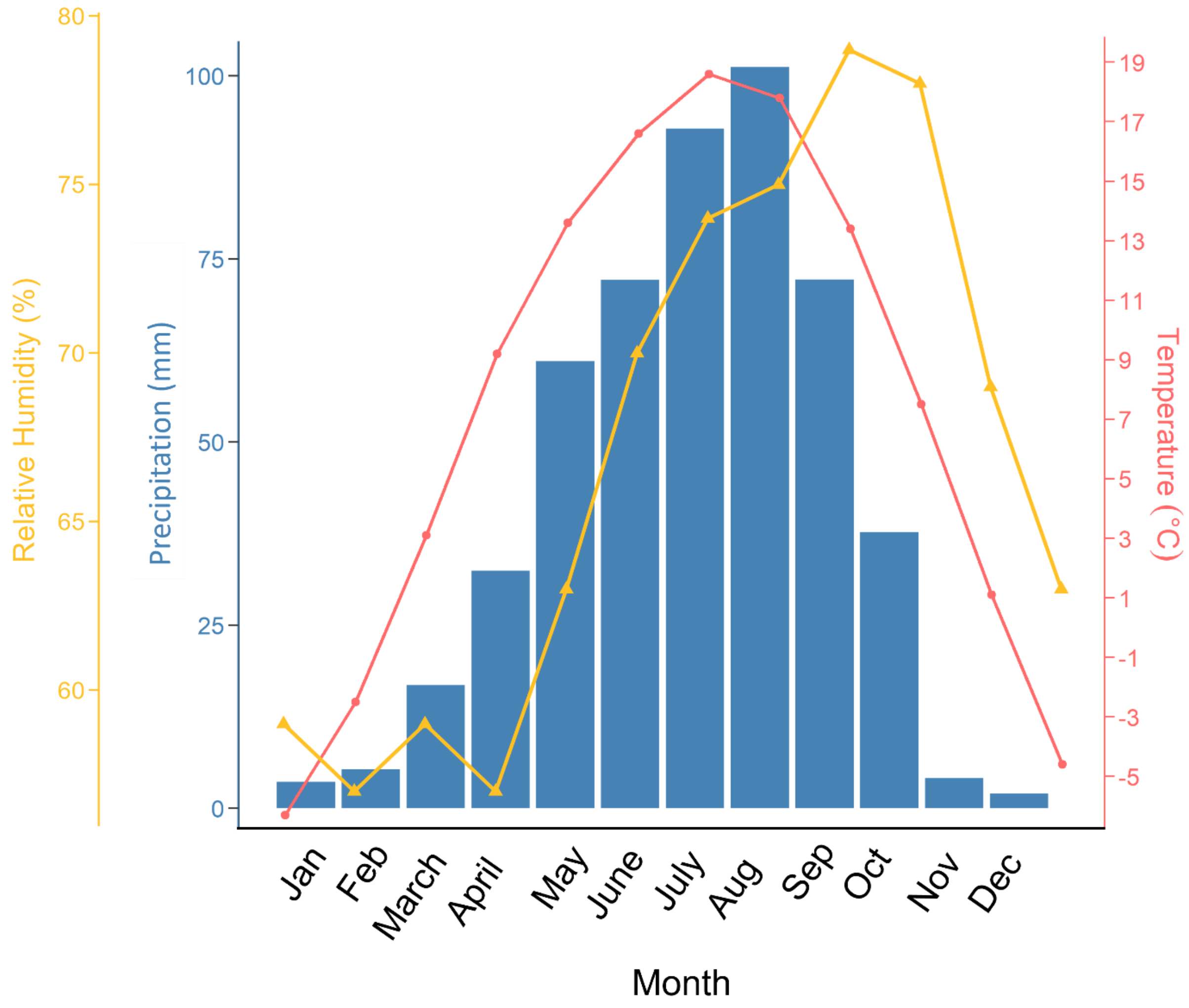

Fig. S4.

Climatological data from Linxia city, north China. Monthly air temperature, monthly precipitation, and monthly relative humidity (RH) at Linxia city between 1981–2010, close to the Yuanbao section on the Chinese Loess Plateau. Data obtained from the China Meteorological Data Service Center, http://data.cma.cn/en.

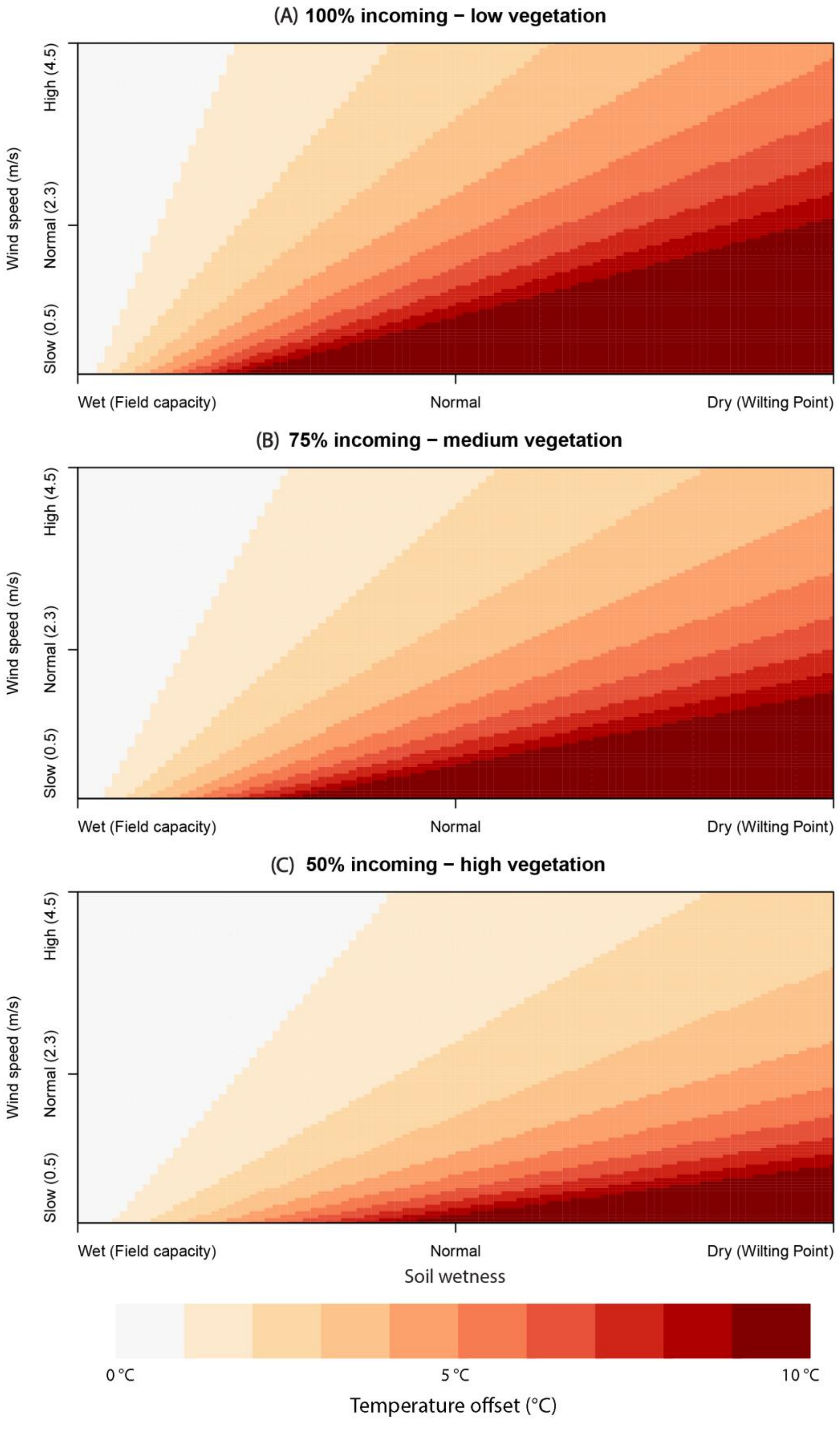
(A) 100% incoming − low vegetation
Wind speed (m/s)
High (4.5)
Normal (2.3)
Slow (0.5)
Wet (Field capacity)
Normal
Dry (Wilting Point)
(B) 75% incoming − medium vegetation
Wind speed (m/s)
High (4.5)
Normal (2.3)
Slow (0.5)
Wet (Field capacity)
Normal
Dry (Wilting Point)
(C) 50% incoming − high vegetation
Wind speed (m/s)
High (4.5)
Normal (2.3)
Slow (0.5)
Wet (Field capacity)
Normal
Dry (Wilting Point)
Soil wetness
0 °C
5 °C
10 °C
Temperature offset (°C)

Fig. S5.

Modelled temperature offsets between soil surface and air temperature. Panels show how different vegetation cover scenarios influence shading and how this affects the temperature offset between soil surface and 2 m air temperature. (A) grass cover (10 cm height) and 0% shading/100% incoming radiation; (B) shrub cover (1 m height) and 25% shading/75% incoming radiation; (C) tree cover (4 m height) and 50% shading/50% incoming radiation.

Data S1. (separate excel file)
Supplementary data for temperature based on brGDGTs, grain size and magnetic susceptibility.

Data S2. (separate excel file)
Supplementary data for temperature based on clumped isotopes of snail shells.

Data S3. (separate R script)
R script for land surface energy partitioning model.
